# Socio-Ecological Hypothesis of Reconciliation: Cultural, Individual, and Situational Variations in Willingness to Accept Apology or Compensation

**DOI:** 10.3389/fpsyg.2020.01761

**Published:** 2020-07-23

**Authors:** Asuka Komiya, Hiroki Ozono, Motoki Watabe, Yuri Miyamoto, Yohsuke Ohtsubo, Shigehiro Oishi

**Affiliations:** ^1^Graduate School of Humanities and Social Sciences, Hiroshima University, Higashihiroshima, Japan; ^2^Faculty of Law, Economics, and Humanities, Kagoshima University, Kagoshima, Japan; ^3^School of Business, Monash University, Subang Jaya, Malaysia; ^4^Department of Psychology, University of Wisconsin-Madison, Madison, WI, United States; ^5^Graduate School of Humanities, Department of Psychology, Kobe University, Kobe, Japan; ^6^Department of Psychology, Columbia University, New York, NY, United States

**Keywords:** apology, compensation, socio-ecological approach, reconciliation, costly signaling theory

## Abstract

The main goal of the present research is to examine socio-ecological hypothesis on apology and compensation. Specifically, we conducted four studies to test the idea that an apology is an effective means to induce reconciliation in a residentially stable community, whereas compensation is an effective means in a residentially mobile community. In Studies 1, 2a, and 2b, American and Japanese participants (national difference in mobility; Study 1) or non-movers and movers (within-nation difference in mobility; Studies 2a and 2b) imagined the situations in which they were hurt by their friends and rated to what extent they would be willing to maintain their friendships upon receipt of apology or compensation. The results showed that compensation was more effective in appeasing residentially mobile people (i.e., Americans and movers) than stable people (i.e., Japanese and non-movers), while apology was slightly more effective appeasing residentially stable people than residentially mobile people (significant in Study 1; not significant in Studies 2a and 2b). In Study 3, by conducting an economics game experiment, we directly tested the hypothesis that mobility would impair the effectiveness of apology and enhance the effectiveness of compensation. The results again partially supported our hypothesis: In the high mobility condition, compensation increased one’s willingness to continue the relationship with the offender, when compared to willingness in the low mobility condition. The importance of socio-ecological perspective on the forgiveness literature is discussed.

## Introduction

Occasional offenses are inescapable in any relationship. Of course, time heals most hurt feelings. Yet, apology or compensation or both is often needed for the victims to fully forgive offensives. Given the importance of the interpersonal reconciliation processes (including apology, compensation, and forgiveness) in the maintenance of relationships, it has been actively investigated in social psychology (Ohbuchi et al., 1989; McCullough et al., 1998; McCullough, 2008; Fehr et al., 2010) in addition to the context of non-human animals (de Waal, 1989) organizations (Bradfield and Aquino, 1999; Aquino et al., 2001), and nations (Long and Brecke, 2003; Hornsey and Wohl, 2013).

The ubiquity of forgiveness across different species points to its evolutionary importance (de Waal, 2000; McCullough, 2008). Psychological research on forgiveness has shown that reconciliatory tendencies and tactics vary substantially across individuals (Howell et al., 2011, 2012) and cultures (Fukuno and Ohbuchi, 1998; Maddux et al., 2011). Although the early evolutionary psychology tends to focus on psychological universals, such as the cheater-detection mechanism (Cosmides, 1989; Tooby and Cosmides, 1992), the growing evolutionary literature suggests that many psychological adaptations are facultative traits (Laland, 2008). That is, people employ adaptive strategies in response to their local environment, such as pathogen level (Low, 1990; Gangestad and Buss, 1993) and sex ratio (Wilson and Daly, 1985; Griskevicius et al., 2012). The aim of the present study is to understand the variation of reconciliatory tendencies in terms of adaptation to socio-ecological environments. Four studies specifically tested whether residential mobility would modify the effectiveness of apology vs. compensation as reconciliation tactics.

### Evolutionary Approach to Reconciliation and Forgiveness

While psychologists (mostly clinical and social psychologists) were accumulating knowledge about the human forgiveness process (Worthington, 2005), animal researchers (mostly primatologists) were conducting research on the reconciliation processes of various species (Aureli and de Waal, 2000). Primatologists do not use the term *forgiveness* because “forgiveness is an internal process to which [researchers] have no access in non-human primates” (de Waal and Pokorny, 2005 p. 18). Accordingly, it is difficult to directly compare the human forgiveness process with an animal reconciliation process. Nevertheless, given the similarity in function (i.e., repair of an endangered relationship), de Waal and Pokorny (2005 p. 18) speculate that both processes probably share an emotional switch that “moves the attitude toward another individual from aggressive and/or fearful to friendly, perhaps even affectionate” and that this psychological mechanism in different species may share a common evolutionary origin.

Although a direct comparison of the human forgiveness process and animal reconciliation process is not feasible, there is some evidence suggesting the presence of commonalities in the two processes. For example, McCullough et al. noted that a prominent hypothesis regarding the function of animal reconciliation (i.e., the valuable relationships hypothesis developed by de Waal, 2000) can be tested in humans (McCullough, 2008; McCullough et al., 2013 for reviews). The valuable relationships hypothesis posits that the function of animal reconciliation is to maintain a valuable relationship that is on the verge of dissolution due to conflicts over less important resources (de Waal, 2000). Accordingly, relationship value is a reliable predictor of animal reconciliation (Cords and Thurnheer, 1993 for experimental evidence from long-tailed macaques). McCullough et al. (2010), for example, tested this hypothesis by assessing the temporal course of forgiveness over 3 months and found that the offender’s relationship value facilitated the rate of forgiveness (see also Burnette et al., 2012; McCullough et al., 2014; Smith et al., 2020 for further evidence for the valuable relationships hypothesis in humans).

Another observable commonality between the human forgiveness process and the animal reconciliation process is the presence of precursory, benign intent signaling (Silk, 2002). When primates reconcile with their former opponent, conciliatory signals from one of the former opponents tend to precede peaceful post-conflict interactions. Similarly, it has been well established that apologies (a human equivalence of a benign intent signal) and other forms of conciliatory gestures (e.g., compensation) facilitate forgiveness (Ohbuchi et al., 1989; Fehr et al., 2010; McCullough et al., 2014 for a meta-analytic review). Given these two commonalities (i.e., the importance of relationship value and conciliatory signals), it is interesting to examine how people react to different types of conciliatory gestures under different socio-ecological environments, where partners’ relationship values vary due to the expected durability of each relationship.

### Apology as an Evolved Reparative Signal

Ohtsubo and Watanabe (2009) proposed an evolutionary model of human apology based on the *costly signaling theory*, which was independently developed in evolutionary biology (Zahavi and Zahavi, 1997) and economics (Spence, 1973). Evolutionary models of signals generally assume the presence of an information asymmetry between two parties (i.e., a signal sender knows something that is not directly knowable by the receiver). If both players will benefit from sharing the accurate information, the receiver does not have to worry about intentional deception (Skyrms, 2010). However, this is no longer the case if the sender benefits from misleading the receiver, because the sender might evolve to send deceptive signals (Krebs and Dawkins, 1984). In the case of reconciliation, an exploitative perpetrator might say “I’m sorry. I won’t do that again,” just to be forgiven and exploit the victim again. Given such a potential conflict of interests, the costly signaling theory predicts that the signal must be sufficiently costly to outweigh the benefit of deception.

Ohtsubo and Watanabe (2009) noted that a costly form of reparative act, such as compensation, could nullify the benefit of exploitation. However, if the perpetrator sincerely wishes to resume the relationship, the cost can be offset by the long-term benefits accruing from the relationship. Therefore, costly compensation signals the honesty of the offender. On the other hand, a minimum form of apology (i.e., just saying “I’m sorry”) does not qualify as a costly signal, and its honesty is not guaranteed. Ohtsubo and Watanabe empirically tested this model, and showed that victims would perceive costly forms of reparative acts (e.g., compensation) as being more sincere than non-costly reparative acts (e.g., a verbal apology). This result was replicated in seven countries (Ohtsubo et al., 2012). Furthermore, a recent functional magnetic resonance imaging study revealed that costly reparative acts engaged the theory-of-mind network in recipients’ brain, suggesting that the recipients read sincere intention from costly reparative acts (Ohtsubo et al., 2018). Therefore, it seems that people have a universal psychological mechanism to assess the honesty of apologizers from the costliness of their reparative acts.

Despite the supportive evidence of the costly apology model, merely saying “I’m sorry” is often sufficient to induce the victim’s forgiveness. In fact, Silk et al. (2000) developed a model of cheap apology and showed that the model nicely fit the observed reconciliatory patterns of female rhesus macaques. Silk et al.’s model assumes that every pair engages in long-lasting interactions (cf. the Ohtsubo and Watanabe model assumes that at least some signalers do not intend to continue interacting with the current partner). Therefore, a one-time deceptive signal permanently deprives the deceiver of the future benefits accruing from the interactions with the victim. Consistent with this model, Silk et al. showed that two female rhesus macaques were more likely to resume peaceful interactions if one of them made quiet calls-non-costly conciliatory gestures-before approaching her former opponent. It is noteworthy that rhesus macaques live in matrilineal groups, and thus neither of the former opponents is likely to leave her natal group (i.e., they are likely to keep interacting with each other in the same group).

A comparison of the two signaling models of apology suggests that non-costly apology is more effective with repeated interactions or in a stable relationship. This is consistent with evolutionary game theoretic analyses. Conducting a computer simulation study, Hruschka and Henrick (2006) found that a forgiving strategy (i.e., letting a few offenses go in established relationships) evolved in an environment in which each player was allowed to develop long-term relationships with a limited number of others. Stability in interpersonal relationships fostered evolution of forgiveness because partner change was costly (e.g., because developing a new relationship was time-consuming). In contrast, in an environment where people can develop relationships with a large number of others, the unconditional forgiveness strategy is not adaptive because it is easily exploited by mobile freeloaders (Enquist and Leimar, 1993). In other words, an unconditional forgiveness strategy, i.e., accepting verbal apologies (e.g., “I’m sorry”), is adaptive in stable environments, whereas a more cautious strategy, which requires a costly form of reparative act, such as compensation, is more adaptive in mobile environments.

### Socio-Ecological Psychology and Reconciliation

Recently, a socio-ecological approach has been applied to systematically investigate the influence of the stability/mobility of one’s social environment (Oishi, 2010; Oishi and Talhelm, 2012; Oishi et al., 2015 for reviews). Although this approach developed relatively independently of the evolutionary psychological approaches to cooperation/reconciliation, its basic ideas and definitions of mobility closely approximate the ones used in evolutionary theories: Residential mobility is defined as the frequency at which people change their residence (Oishi, 2010).

Previous studies suggest that residential mobility affects people’s social network, in particular the expected length of relationships (Oishi, 2010; Yuki and Schug, 2012). More specifically, individuals who live in a residentially stable environment expect to interact with the same group of individuals for an extended period of time, whereas individuals who live in a residentially mobile environment expect to interact with the same group of individuals only for a short period of time. Moreover, consistent with the aforementioned evolutionary model by Enquist and Leimar (1993), recent studies have shown that people are more cooperative toward their groups in stable environments than in mobile environments (Oishi et al., 2007b, 2009).

Applying the notion of residential mobility to the evolutionary models, we predicted that verbal (no-cost) apology is more effective in residentially stable environments, while compensation is more effective in residentially mobile environments. Although many studies have tested the effectiveness of verbal apologies (Fehr et al., 2010) and compensation (Desmet et al., 2010, 2011) separately, or compared their relative effectiveness without controlling for the relevant socio-environmental variables (Bottom et al., 2002; Ohtsubo and Watanabe, 2009; Komiya et al., 2018; Ohtsubo, 2020) to our knowledge, no study has examined the relationship between residential mobility and acts inducing reconciliation.

However, there is suggestive evidence. As many researchers point out (Yuki et al., 2007; Oishi, 2010; Yamagishi, 2011) there is a substantial difference in residential mobility between the United States and Japan: Almost half of Americans moved between 1995 and 2000 (Schmitt, 2001) while 28.1% of Japanese moved during the same years (Statistics Bureau and Statistics Center of Japan). Thus, based on the above arguments, it is expected that a verbal apology is more effective in Japan than in the United States. Fukuno and Ohbuchi (1998) scenario experiment showed that this was actually the case: An apology offered by the offender was more effective in improving Japanese participants’ impressions of the offender than in improving Americans’. Ohbuchi et al. (2009) also showed that Japanese respondents were more satisfied with the offender’s apology than American ones were. Moreover, studies of perpetrators’ account strategies have also revealed a societal difference mirroring the tendency of victims: Japanese are more likely to apologize than Americans (Hamilton and Hagiwara, 1992; Itoi et al., 1996). In past research, such variations have usually been explained by differences in cultural values such as placing importance on social harmony or relational concerns. Moreover, no study has examined whether compensation is more effective in the United States than in Japan. In contrast, this research is the first attempt to explain this cultural difference in effective conciliatory tactics, if such really exists, by the difference in a socio-ecological factor-residential mobility.

### The Present Studies

#### Overview

To fill the lack of empirical evidence for the socio-ecological hypothesis regarding the relationship between residential mobility and reconciliation, we conducted four studies, all of which employed the same operationalization of reconciliation-the victim’s willingness to maintain the relationship with the perpetrator. On the other hand, these four studies utilized *different* operationalizations of mobility. Study 1 tested the cross-national prediction: Apology would be more effective in Japan than in the United States, whereas compensation would be more effective in the United States than in Japan. Study 2a (Japan) and Study 2b (United States) tested the within-nation prediction: Apology would be more effective in inducing reconciliation from non-movers than frequent movers, whereas compensation would be more effective in inducing reconciliation from frequent movers than non-movers. Because Studies 1, 2a, and 2b were correlational studies, the causal role of residential mobility cannot be established. To address this limitation, we follow previous socio-ecological research (Oishi et al., 2012; Yuki et al., 2013) that used a similar logic and the combination of correlational studies and an experiment. Specifically, Study 3 directly manipulated mobility and tested whether an environment associated with frequent partner change (i.e., high mobility) would make apology less effective and compensation more effective as a reconciliatory tool.

#### Sample Size Determination and *post hoc* Power Analysis

Because there were no studies investigating the effectiveness of reconciliatory tactics at three different levels (i.e., at the national, individual, and situation levels), we could not accurately estimate the effect size of each study. We thus determined the sample size with reference to conventional cross-cultural research conducted in the past (Komiya et al., 2011). Specifically, we decided to collect at least 40 participants per cell throughout the four studies. We then conducted a *post hoc* power analysis to calculate the power of each study (see section “A *Post Hoc* Power Analysis”).

## Study 1: A United States–Japan Comparison Study

Study 1 aimed to expand the previous cross-cultural research on reconciliation by examining the effect of compensations (i.e., a costly reparative act), in addition to the effect of apology (i.e., non-costly reparative act). We predicted that apology would be more likely to promote reconciliation in Japan than in the United States, whereas compensation would be more effective for reconciliation in the United States than in Japan.

### Methods

#### Participants

Fifty-one European American undergraduates at the University of Wisconsin, Madison (24 men and 27 woman) and 50 Japanese undergraduates at Waseda University (37 men and 13 women) participated in this study. They received course credits in exchange for their participation.

#### Procedure

Participants completed a packet of questionnaire that included three scenarios (Musical, Travel, and Book) with four offender behaviors. All scenarios are presented in Supplementary Material. Participants first read a scenario in which an offender caused one of his/her friends trouble. For example, the Book scenario described a situation in which an offender borrowed a book from his friend and stained it by mistake. After each scenario, they read all four possible behaviors of the offender: Neither apology nor compensation (NN condition; saying nothing and not giving compensation), apology without compensation (AN condition; e.g., saying “I’m sorry for staining the book”), compensation without apology (NC condition; e.g., buying a new book and returning it to his friend), and compensation with apology (AC condition; e.g., saying “I’m sorry for staining the book” and buying a new book and returning it to his friend). The scenarios and the perpetrator’s behaviors were fixed in the aforementioned order. The questionnaire was translated by two Japanese-English bilinguals using the back-translation method to confirm consistency between cultures.

For each behavior, assuming that the participants had been victims, they rated to what extent they would hope to remain friends with the offender on a seven-point scale, ranging from 1 (definitely not) to 7 (definitely). Because participants’ ratings for the three scenarios were correlated with each other within each of the four within-participant conditions (Cronbach’s α = 0.80, 0.75, 0.87, and 0.65, for NN, AN, NC, and AC, respectively), we collapsed the three scenarios. In particular, we averaged the three willingness-to-maintain-friendship scores within each condition and used the four aggregated scores in the following analyses. Therefore, each participant had four willingness-to-maintain-friendship scores corresponding to the four within-participant conditions.

### Results and Discussion

**FIGURE 1:**
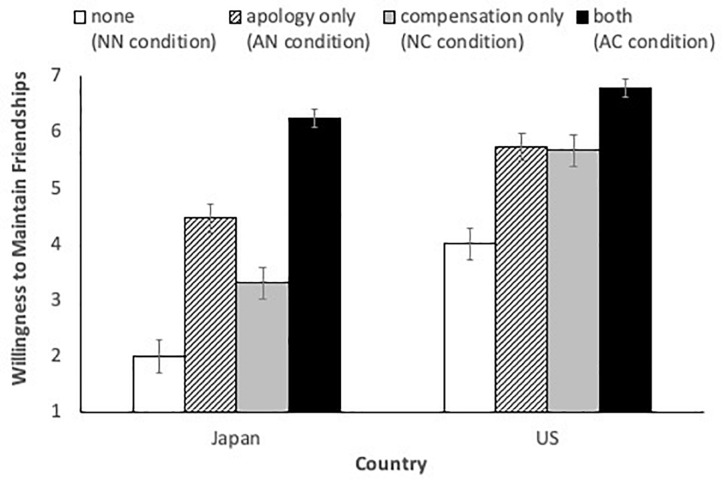
The means and 95% confidence intervals (95% CI) of willingness to maintain friendships in each condition (Study 1). Error bars represent 95% CIs.

The means and 95% confidence intervals (95%CIs) of the willingness to maintain friendship are shown in Figure 1. A 2 (apology: NN+NC or AN+AC) × 2 (compensation: NN+AN or NC+AC) × 2 (nation: United States or Japan) mixed design ANOVA on the willingness-to-maintain-friendship score revealed the significant three-way interaction, *F*(1,99) = 17.86, *p* < 0.001, partial η^2^ = 0.15. Thus, we moved to the analyses which specifically focused on our concerns. Our central prediction was that apology would be more effective than compensation in Japan, whereas compensation would be more effective than apology in the United States. In other words, we hypothesized that (i) both Japanese and Americans would be more willing to reconcile with the offender in the AN condition than in the NN condition, but the apology effect would be larger for Japanese than Americans, (ii) both Japanese and Americans would be more willing to reconcile with the offender in the NC condition than in the NN condition, but the compensation effect would be larger for Americans than Japanese, and (iii) both Americans and Japanese would be more willing to reconcile with the offender in the AC condition than in the NN condition, and the size of the effect would not differ between United States and Japan^1^.

To test the first hypothesis regarding the role of apology, we conducted a 2 (apology: NN or AN) × 2 (nation: United States or Japan) mixed design ANOVA on the willingness-to-maintain-friendship score (Figure 1; white vs. diagonal bars). As expected, both Americans and Japanese were more willing to reconcile with the apologizing offender than non-apologizing offender, *F*(1,99) = 474.35, *p* < 0.001, partial η^2^ = 0.83. More importantly, this effect of apology was qualified by the apology-nation interaction, indicating that the effectiveness of apology was stronger for Japanese than for Americans *t*(49) = 18.50, *p* < 0.001, *d* = 2.64 for Japanese and *t*(50) = 12.45, *p* < 0.001, *d* = 1.69 for Americans. In addition, although we did not predict, the main effect of nation was significant, *F*(1,99) = 93.58, *p* < 0.001, partial η^2^ = 0.49, showing that Americans were generally more willing than Japanese to reconcile with offenders.

Next, we tested the second hypothesis regarding the role of compensation using a 2 (compensation: NN or NC) × 2 (nation: United States or Japan) mixed design ANOVA (Figure 1; white vs. gray bars). Again, as expected, the main effect of compensation was significant: Both Americans and Japanese were appeased by the offender’s offering compensation, *F*(1,99) = 209.86, *p* < 0.001, partial η^2^ = 0.68. However, the predicted interaction between compensation and nation remained marginally significant, although the effect of compensation was slightly stronger among Americans, *t*(50) = 12.15, *p* < 0.001, *d* = 1.63, than among Japanese *t*(49) = 8.54, *p* < 0.001, *d* = 1.22. In addition, the main effect of nation was again significant, *F*(1,99) = 146.46, *p* < 0.001, partial η^2^ = 0.60.

Finally, we tested the third hypothesis by a 2 (apology: NN or AC) × 2 (nation: United States or Japan) mixed design ANOVA (Figure 1; white vs. black bars). Again, as expected, the main effect of apology and compensation was significant: Both Americans and Japanese were appeased by the offender’s apology with compensation, *F*(1,99) = 891.86, *p* < 0.001, partial η^2^ = 0.89. Moreover, the main effect of nation was significant, *F*(1,99) = 123.84, *p* < 0.001, partial η^2^ = 0.56. Unexpectedly, there was a significant interaction effect, *F*(1,99) = 36.11, *p* < 0.001, partial η^2^ = 0.27. As seen in Figure 1, although both Japanese and American participants were more willing to reconcile with the offender who provided apology and compensation than the offender who did not provide any apology or compensation; *t*(49) = 25.72, *p* < 0.001, *d* = 5.46 (Japanese) and *t*(50) = 15.33, *p* < 0.001, *d* = 3.08 (Americans), the difference between the AC and NN conditions was smaller for Americans than for Japanese. This unexpected result may be due to the fact that Americans were far more willing to reconcile with those who did not provide any apology or compensation than were Japanese, *t*(90.02) = 9.75, *d* = 1.94. Thus, the observed interaction appears to be driven by the American tendency to forgive the offenders who did not provide any apology or compensation.

Overall, the obtained data fit our hypotheses quite well. Apology was a more effective means of relationship maintenance for Japanese compared to for Americans, whereas compensation was a more effective means for Americans than for Japanese.

It should be noted that, however, in this study, Americans were more likely to forgive offenders who did not conduct any reconciliatory acts. That is, the baseline was different between nations. This could be because Americans tend to maintain larger social networks in general than others (Oishi, 2010) or due to the American’s tendency to be more positive than Japanese (Hamamura et al., 2009). Thus, it is possible that Americans judged offenses as being less severe and are more likely to remain friends even though offender’s provided nothing. Importantly, the different level of the baseline could lead to not only the unexpected interaction effect (regarding the third hypothesis), but also the expected interaction effect of nation and apology (regarding the first hypothesis). Given that it possibly comes from a cultural difference, a within-nation analysis could be one way to address this ambiguity.

## Study 2A: An Examination of the Individual Difference-The Case of Japan

Study 1 showed the expected differences between Japanese (low mobility) and Americans (high mobility) in the effectiveness of apology and compensation. However, the cross-nation comparison is vulnerable to various alternative explanations, as Japan and the United States are different not just in residential mobility rates, but also other factors such as language, moral education, religion, and history. Also, we found the baseline difference which could be associated with cultural differences of psychological tendency. If our socio-ecological hypotheses are correct, we should find parallel differences within each nation. Thus, in Studies 2a (Japan) and 2b (United States), we investigated within-nation variations in the effectiveness of apology and compensation. The conceptual replication of Study 1 in each country excludes most of the alternative explanations, such as language, education, and religion. In addition, we did not measure residential mobility of our samples in Study 1. Therefore, strictly speaking, we cannot ascertain whether Japanese undergraduates in fact experienced less moves than American undergraduates. In Study 2, to confirm this assumption, we compared the number of moves that Japanese undergraduates (Study 2a) and American undergraduates (Study 2b) experienced.

### Methods

#### Overview

In Study 2a, we tested (i) whether residentially stable Japanese would be appeased more by apology than residentially mobile Japanese and (ii) whether residentially mobile Japanese would be appeased more by compensation than residentially stable Japanese. To investigate these hypotheses, we analyzed the unpublished data which was collected for another purpose (Komiya et al., 2018)^2^. In the study, all participants read two scenarios, were exposed to either the offender’s verbal apology or compensation for the scenarios (thus, unlike in Study 1, conciliatory act was manipulated as a between-participants factor), and reported the willingness to remain friends with the offender. We tested the model in which the willingness to remain friends as the dependent variable and participants’ move experience and the reconciliatory act condition as the primary predictor variables.

#### Participants

One hundred and eighty-five Japanese undergraduates at Kobe University (95 men, 89 women, one unknown) participated in our study in exchange for 500 Japanese yen (roughly 5 USD).

#### Procedure

Participants were invited to the laboratory and asked to fill out the questionnaires. The questionnaire included two scenarios: a Book Scenario and a Baseball Scenario (for details, see Komiya et al., 2018). The scenario order was counterbalanced.

Participants first read a scenario in which one of his/her friends caused a participant trouble. After each scenario, assuming that he/she had been the victim, participants rated to what extent they would want to remain friends with the offender on a six-point scale ranging from 1 (definitely not) to 6 (definitely). This rating was used as the baseline. The participants then read the scenarios in which they received either apology or compensation from the offender. Participants again rated how likely they were to remain friends with the offender on a six-point scale. Since the ratings across the two scenarios were correlated (*r* = 0.42 for the baseline; *r* = 0.53 for the target item), the scenario factor (a within-participant factor) was collapsed for the following analyses (i.e., each participant had a single baseline reconciliation score and a single target reconciliation score).

After finishing the rating, participants were asked to provide all of the residential moves they experienced. We counted the frequency of moves across cities when participants were between 5 and 18 years old (Oishi et al., 2007a). According to this criterion, 114 (62.3%) had never moved, 46 (25.1%) had moved once, 15 (8.2%) moved twice, and 8 (4.4%) moved three times (*M*_*move*_ = 0.6 times, *SD* = 0.82). Also, participants provided the information about the current residential status, choosing from living alone (*n* = 94), with family (*n* = 80), or other (e.g., with relatives, *n* = 7). Because the latter variable (i.e., residential status) may influence the estimated continuity of relationships, we also controlled for this factor. Four people failed to provide the moving and residence information, thus leaving us with 181 participants in the following analyses.

### Results and Discussion

Incorporating the baseline rating, residential status (“with family or not” and “living others or not”), the number of moves, conciliatory acts (apology vs. compensation), and the number of moves × conciliatory acts interaction as independent variables, a multiple regression analysis was conducted on the reconciliation score (Table 1). Since the model included an interaction term, all variables were mean-centered. The entire model explained the 55% of the variance in the willingness-to-maintain-friendship score, *F*(6,174) = 36.05, *p* < 0.001, *R*^2^ = 0.55. As shown in Table 1, the results showed a trend for the mobility × conciliatory acts interaction. Simple slope tests revealed that whereas there was no significant effect of moving experiences on the apology effectiveness, *b* = −0.05, *SE* = 0.10, β = −0.03, *t*(174) = −0.44, *p* = 0.66, 95% CI [−0.26, 0.17], partial *r*^2^ = 0.001, there was an effect of moving experiences on the compensation effectiveness, *b* = 0.22, *SE* = 0.10, β = 0.15, *t*(174) = 2.18, *p* = 0.030, 95% CI [0.02, 0.42], partial *r*^2^ = 0.027. That is, frequent movers were more willing to reconcile with the offenders who provided compensation than infrequent movers.

**TABLE 1:** The results of regression analysis (Study 2a).

**Variables**	***b* (*SE*)**	**β**	***t*(174)**	***p***	**95% CI**
**Control variables**					
Baseline	0.60(0.06)	0.51	10.07	**<0.001**	[0.49, 0.72]
Living with family	0.02(0.13)	0.10	0.19	0.849	[−0.22, 0.27]
Living with someone (not family)	0.71(0.32)	0.11	2.21	**0.029**	[0.07, 1.34]
**Independent variables**					
Conciliatory acts (apology = −1, compensation = 1)	−0.64(0.06)	–0.54	–10.55	**<0.001**	[−0.76, −0.52]
Number of moves	0.09(0.07)	0.06	1.15	0.252	[−0.06, 0.23]
Moves × conciliatory acts	0.13(0.07)	0.09	1.80	0.073	[−0.01, 0.28]

*The bold values mean statistical significance at 5% level.*

The results supported one of our hypotheses: frequent movers were more willing to reconcile with the offenders who provided compensation than infrequent movers were (cf. the second hypothesis in Study 1). However, infrequent movers were no more appeased by apology than frequent movers (cf. the first hypothesis in Study 1). This insignificant result might be attributable to the small variance in the number of moves in Japan. More than half of the participants (62.3%) in this study did not experience any moves. This relatively small variation might not be enough to find the individual difference of preference for apology. We addressed this issue in Study 2b.

## Study 2B: An Examination of the Individual Difference—The Case of United States (Take 2)

Study 2b is a close replication of Study 2a in United States. where the variation in moving experiences among undergraduates would be larger (Schmitt, 2001). In addition, we included ethnicity, i.e., cultural background, as an independent variable in Study 2b. As discussed in Section “Introduction,” some studies report the cross-national difference in the effectiveness of apology based on the endorsement of social harmony in the collectivistic cultural contexts (Fukuno and Ohbuchi, 1998; Ohbuchi et al., 2009). Also, more directly Fehr and Gelfand (2010) found that those who emphasized the independent self-construal (i.e., European Americans’ cultural tradition) were appeased more by compensation, while those who emphasized relational or collective self-construal (i.e., other Americans’ cultural tradition) were appeased more by other cost-free forms of apology. Even within the United States, those who have collectivistic cultural backgrounds may exhibit slightly different patterns than those who have individualistic cultural backgrounds. We thus tested whether ethnicity would affect the effectiveness of apology and compensation (i.e., the ethnicity × offender’s behavior interaction) and whether it would interact with moving experiences (i.e., the ethnicity × offender behavior’s × move interaction).

### Methods

#### Participants

Two hundred and thirty-nine undergraduates at University of Virginia (100 men, 139 women) participated in our study in exchange for a partial course credit. Because international students (*n* = 19) and participants who refused to write down their moving experiences (*n* = 14) were excluded from the analyses below, the final sample size was 208 (128 Caucasian Americans, 46 Asian Americans, 13 African Americans, two Hispanic/Latinos, and 20 mixed-racial).

#### Procedure

The procedure was the same as Study 2a except that the variation of each scenario was not used (see Supplementary Material; see also footnote 2). Since the ratings were correlated across the scenarios (*r* = 0.45 for the baseline; *r* = 0.49 for the target item), they were averaged as the baseline score and the reconciliation score, respectively. One hundred and fourteen (54.8%) had never moved, 55 (26.4%) had moved once, 24 (11.5%) moved twice, and 15 (7.2%) moved three times and more (maximum: six times, *M*_*move*_ = 0.78 times, *SD* = 1.16). As expected, this sample experienced more moves than the sample of Study 2a, *t*(373.23) = 2.34, *p* = 0.02, *d* = 0.23 and had larger variation, *F*(1,387) = 7.16, *p* = 0.008 (Levene’s test): The number of experiences of moves ranged from 0 to 3 in Study 2a (Japan), while it ranged from 0 to 6. Given the large variation and skewed distribution of residential mobility (Skewness = 1.98 and Kurtosis = 4.37), we applied the square-root transformation to the number of moves before conducting the reported analyses.

### Results and Discussion

As in Study 2a, a multiple regression analysis was conducted on the reconciliation score, entering the baseline score (as a control variable), offender’s behavior (apology vs. compensation), ethnicity (Caucasians vs. others), the number of residential moves, and the interaction terms across the variables (i.e., offender’s behavior × moves, moves × ethnicity, ethnicity × offender’s behavior, offender’s behavior × ethnicity × moves) into the equation. Since the model included the interaction terms, all variables were mean-centered. The entire model explained the 57% of the variance in the relationship maintenance score, *F*(8,199) = 33.21, *p* < 0.001, *R*^2^ = 0.57 (Table 2). As shown in Table 2, most importantly, the mobility × offender’s behavior interaction was significant. The simple slope tests showed that whereas moving experiences did not affect the effectiveness of apology, *b* = −0.06, *SE* = 0.11, β = −0.04, *t*(199) = −0.56, *p* = 0.57, 95% CI [−0.28, 0.15], partial *r*^2^ = 0.002, it increased the effectiveness of compensation, *b* = 0.24, *SE* = 0.10, β = 0.15, *t*(199) = 2.34, *p* = 0.020, 95% CI [0.04, 0.44], partial *r*^2^ = 0.027. That is, replicating Study 2a, frequent movers were more willing to reconcile with the offenders who provide compensation than infrequent movers, whereas there was no effect of residential mobility on the effectiveness of apology. This interaction was not qualified by ethnicity: The ethnicity × offender’s behavior × mobility interaction was not significant (Table 2).

**TABLE 2:** The results of regression analysis (Study 2b).

**Variables**	***b* (*SE*)**	**β**	***t*(199)**	***P***	**95% CI**
**Control variables**					
Baseline	0.72(0.05)	0.72	14.84	**<0.001**	[0.63, 0.82]
**Independent variables**					
Conciliatory acts (apology = −1, compensation = 1)	−0.12(0.05)	–0.12	–2.41	**0.017**	[−0.22, −0.02]
Ethnicity (Caucasian = −1, other ethnicity = 1)	−0.08(0.06)	–0.07	–1.42	0.157	[−0.19, 0.03]
Number of moves	0.09(0.07)	0.06	1.19	0.235	[−0.06, 0.23]
Move × conciliatory acts	0.15(0.07)	0.10	2.00	**0.046**	[0.002, 0.30]
Ethnicity × conciliatory acts	−0.10(0.06)	–0.09	–1.91	0.058	[−0.21, 0.003]
Move × ethnicity	0.08(0.08)	0.05	1.02	0.307	[−0.08, 0.24]
Move × ethnicity × conciliatory acts	0.06(0.08)	0.04	0.75	0.452	[−0.10, 0.22]

*The bold values mean statistical significance at 5% level.*

In addition, the regression analysis revealed a trend for the ethnicity × offender’s behavior interaction (Table 2). Simple slope tests showed that there was no significant effect of ethnicity on the effectiveness of apology, *b* = 0.03, *SE* = 0.08, β = 0.02, *t*(199) = 0.34, *p* = 0.74, 95% CI [−0.23, 0.19], partial *r*^2^ = 0.001, but a significant effect on the effectiveness of compensation, *b* = −0.18, *SE* = 0.08, β = −0.16, *t*(199) = −2.36, *p* = 0.019, 95% CI [−0.34, −0.02], partial *r*^2^ = 0.027, showing that Caucasian Americans were more likely to reconcile with the offenders who provided compensation than were non-Caucasian participants.

Overall, Study 2b replicated the results of Study 2a: Frequent movers were more appeased by compensation than infrequent movers were. Also, the effect of residential mobility on compensation was not qualified by ethnicity. Even after controlling for the individual’s ethnic background, residential mobility was associated with the effectiveness of compensation. On the other hand, infrequent movers were no more appeased by an apology than frequent movers, even though there was a relatively large variation in moving experiences. This might be due to the ambiguous nature of the scenario study. That is, different participants might have interpreted the offender’s apology in different ways-some might have taken it as merely saying “sorry” (as we intended), whereas others might have assumed that the offender exhibited a full-fledged conciliatory gesture involving not only a verbal apology but other elements as well, such as an expression of remorse and acknowledgment of responsibility. This possibility is addressed in Study 3, in which we manipulated the apologetic message as a part of the experimental manipulation. We further discuss possible reasons for this result in Section “General Discussion.”

## Study 3: An Experimental Study

Studies 1, 2a, and 2b provided strong support for our hypotheses regarding compensation and partial support for our hypotheses regarding verbal apology. Because these studies were correlational, the causal role of residential mobility was not established. To address this limitation, in Study 3, we directly manipulated the expected length of interactions and examined how people would evaluate apology and compensation. To this end, we employed a noisy version of the trust game (Berg et al., 1995; Ho, 2012 for the noisy-trust game) that was played by two players. Using this game, we manipulated the experimental analog of residential mobility, which was the key independent variable of Studies 1, 2a, and 2b; approximately half of participants expected to play the game with the same partner for multiple rounds (i.e., low mobility), while the other half expected frequent partner changes (i.e., high mobility). We expected that when participants played with the same partner for a relatively long period of time, they would be willing to stay with their apologizing partner (i.e., an offender who says “I’m sorry” but does not offer any compensation). On the other hand, when their partners changed frequently, people would be less willing to keep interacting with their offender unless the offender offered compensation.

### Methods

#### Participants

Ninety-eight Japanese undergraduate and graduate students at Waseda University (60 men, 38 women, mean age = 20.53, range = 18–32) participated in this study. They were recruited through a flyer posted on a university’s portal website. For each experimental session, 10–20 students were invited to the laboratory. Participants in three sessions (*n* = 52) were assigned to the high-mobility condition, whereas those in other three sessions (*n* = 46) were assigned to the low-mobility condition. Due to a computer program error, four participants in the high-mobility condition failed to finish the last round. We included the data of these participants in the analyses (removing their data did not affect any results).

#### Trade Game (Noisy Trust Game With Apology)

**FIGURE 2:**
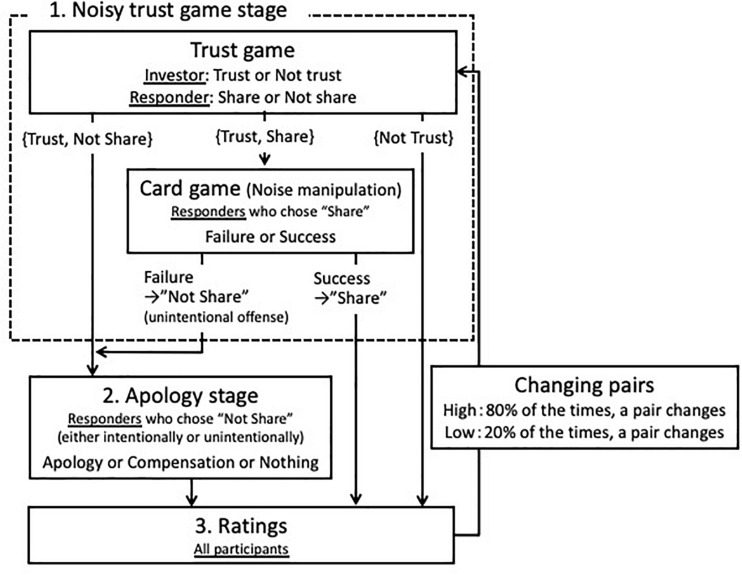
A sequence of trade game. Further explanation is shown in Supplementary Material.

Participants played a series of noisy trust games with the apology option, which we called the “trade game” in the experiment. The program was developed by using the WebMatrix platform (Microsoft©). The game consisted of three sections: (1) the noisy trust game (surrounded by broken line in Figure 2), (2) the apology stage, and (3) ratings about the willingness to maintain the relationship. At the beginning of each round, participants were informed of whether they would play this round with the same partner as the one in the previous round.

##### Noisy trust game stage

In the noisy trust game, participants were randomly paired, assigned to either an investor or a responder role, and played a modified version of the trust game: Both the investor and the responder received an initial endowment of 30 JPY (approximately 0.3 USD). The investor first decided whether to entrust their initial endowment to the responder. If the investor chose the “trust” option, the responder received a tripled amount of the entrusted resource (90 JPY) and then decided whether to share the total resource of 120 JPY equally with the investor. This standard form of the trust game was modified to create the ambiguity of the responder intention: In the “noisy” game, approximately a third of the cooperative choices of the responders were experimentally altered to the uncooperative choices in a random manner. Therefore, when the investors learned that their responders did not share the resource, they could not be certain about their responders’ intention (i.e., the responders might have chosen the share option but their good intention might not have been effectuated). The detailed procedure is shown in Supplementary Material.

##### Apology stage

When the responder failed to share the resource either intentionally or unintentionally, the game went on to the apology stage. In this stage, the non-sharing responder was given three options: “apology,” “compensation,” and “doing nothing.” When the responder chose the apology option, an apology message (i.e., I am sorry to have chosen “not to share”) was sent to the partner. When the investor chose the compensation option, the investor’s initial endowment was recovered and both players received 30 JPY. When the investor chose the “doing nothing” option, the responder took all of the resources, and the investor was simply informed that he/she would receive 0 JPY on this round.

After all the decisions had been made, participants were informed of the results of the trade game. The investor’s choice (i.e., “to trust” or “not to trust”), the responder’s choice corrected by the result of card game (i.e., “to share” or “not to share”), and the responder’s choice in the apology stage if applicable (i.e., “apology,” “compensation,” and “doing nothing”) were explicitly shown on the investor’s computer display. As for the responder’s choice, the investor could not know whether the responder chose not to share intentionally (i.e., choosing not to share in the trust game) or accidentally (i.e., choosing a “failure” card in the card game). Both the investor and the responder knew about the card game and the possibility of noise in the game, and both parties were aware of the other player’s knowledge of the noise.

##### Ratings

At the end of each round, participants rated (i) the likelihood of trusting the same partner on the next round if they would play the game as an investor and (ii) their preference for playing the game with the same partner on the next round. To assess how likely the intention to maintain the relationship (corresponding to maintaining friendships in Studies 1, 2a, and 2b), we averaged these two items (*r* = 0.58). This was our main dependent variable, *maintaining relationships*, for Study 3. As a manipulation check, they also answered their subjective assessment of the likelihood of being paired with the same partner on the next round. All these variables were measured on a seven-point scale, ranging from “1: disagree very much (or very unlikely)” to “7: agree very much (or very likely).”

#### Procedure

On arrival, participants were ushered to separate cubicles, each equipped with a computer. After completing the informed consent forms, they first read the rules of the trade game. They were then assured that they would receive a sum of money contingent on the score they would accumulate throughout the trade game. Participants then received ID numbers that enable them to anonymously interact with other participants. They were then explained how frequently interaction partners would change in the game. In the high-mobility condition, interaction partners changed 80% of the time after each round, whereas the likelihood was 20% in the low-mobility condition. Participants then played the 20 rounds of the trade game (participants were kept unaware of how many rounds they would play the game). After finishing the 20 rounds, participants completed a post-game questionnaire including some manipulation check items. They were then debriefed and paid their rewards contingent on their game scores.

### Results and Discussion

#### Manipulation Check

We first checked the effectiveness of the partner mobility manipulation. Because each participant rated the likelihood every round, we averaged the ratings across 20 rounds and used the aggregated score in this analysis. A *t*-test indicated the manipulation was successful: Participants in the low-mobility condition were more likely to assess the interaction would continue in the next trial than participants in the high-mobility condition, *M*_*low*_ = 4.90 (SD = 1.07) vs. *M*_*high*_ = 2.87 (SD = 0.89), *t*(96) = 10.28, *p* < 0.001, *d* = 2.06.

#### Trust, Reciprocal Reaction, and Conciliatory Acts

First, we compared a general level of cooperation (i.e., entrusting one’s endowment and sharing the entrusted resource) between the low- and the high-mobility conditions. For each participant, the trust rate was obtained by dividing “the number of trust decisions he/she made” by “the number of rounds in which he/she played the investor role.” Similarly, the sharing rate was obtained by dividing “the number of sharing decisions he/she made” by “the number of rounds in which he/she had been trusted by his/her partner.” The mean trust rate and sharing rate are shown in Table 3. Consistent with evolutionary and socio-ecological theorizations, low mobility fostered cooperation: Both the trust rate and sharing rate were significantly higher in the low-mobility condition than they were in the high-mobility condition, *t*(96) = 4.99, *p* < 0.001, *d* = 1.00 for trust and *t*(92.23) = 3.87, *p* < 0.001, *d* = 0.78 for sharing. As a result, participants in the low-mobility condition received a larger monetary reward amount than those in the high-mobility condition, *t*(96) = 3.35, *p* = 0.001, *d* = 0.67 (Table 3). These results were consistent with the assumptions of our hypothesis.

**TABLE 3:** The means (SDs) of cooperation rate, the monetary earning, and the ratio of conciliatory acts by the mobility condition.

	**Choice (%)**		**Earnings**	**Conciliatory acts (%)**		
**Mobility**	**Trust**	**Share**	**(JPY)**	**Apology**	**Compensation**	**Nothing**
Low	71.85 (29.84)	65.77 (33.92)	950.87 (216.88)	45.98 (40.61)	40.81 (41.17)	13.21 (30.05)
High	40.39 (32.53)	35.87 (41.38)	816.92 (178.78)	48.04 (45.22)	37.25 (45.08)	14.71 (34.30)

We then analyzed the frequency of different types of conciliatory acts. For each participant, the rates of apology, compensation, and do-nothing were computed by dividing “the number of each choice he/she made after unintentional offenses” by “the number of unintentional offenses he/she committed^3^.” A series of *t*-tests showed that the mean apology, compensation, and do-nothing rates did not significantly differ between the high- and low-mobility conditions, *t*s < 1, *p*s > 0.77 (Table 3).

#### Hypothesis Testing: Reaction to Apology and Compensation

In this section, we analyzed participants’ *maintaining relationships* after their partner’s “not share” decision, that is, how much participants were willing to forgive their partner’s transgression. Although all participants played 20 rounds of the trust games, the number of rounds in which their partner had chosen “not share” varied across participants. Moreover, each participant responded to variable numbers of apology, compensation, and do-nothing instances. Furthermore, the maintaining relationship scores were nested within individual, while the manipulation of mobility was at the level of between-individual^4^. Thus, we tested our hypothesis using Mplus 4.21’s multilevel model (Muthén and Muthén, 2007). Specifically, the model was as follows:

Level 1 (within-individual)
DV = *b*_0_ + *b*_1_^∗^dummy1 + *b*_2_^∗^dummy2 + error,

where DV = intention to stay in the relationship with the current partner (forgiveness), dummy 1 was coded as 1 = “compensation”; 0 = “do nothing” or “apology,” and dummy 2 was coded as 1 = “apology”; 0 = “do nothing” or “compensation.” In this dummy coding, “do nothing” was the reference group, and the dummy variable 1 [dummy variable 2] tested whether a participant was more willing to stay with the current partner when the partner compensated [apologized] than when the partner did not do anything. The intercept from this model indicated the degree of willingness to stay in the relationship with the current partner when the current partner did not do anything (i.e., baseline intention to remain in the relationship).

Level 2 (between-individual) model was as follows:
*b*_0_ (Level 1 intercept) = *r*_00_ + *r*_01_^∗^mobility + *r*_02_^∗^gender + error
*b*_1_ (compensation slope) = *r*_10_ + *r*_11_^∗^mobility + *r*_12_^∗^gender
*b*_2_ (apology slope) = *r*_20_ + *r*_21_^∗^mobility + *r*_22_^∗^gender,

where the mobility condition was coded as 0 = low mobility; 1 = high mobility, and gender was coded as 0 = female; 1 = male.

As expected, participants were more willing to stay in the relationship with the current partner when the partner compensated than did nothing, *r*_10_ = 2.825, *SE* = 0.423, *z* = 6.681, *p* < 0.001. They were also more willing to stay in the relationship when the partner apologized than did nothing, *r*_20_ = 0.686, *SE* = 0.250, *z* = 2.744, *p* = 0.006.

Unexpectedly, when the partner did not do anything, participants in the low mobility condition were more willing than those in the high mobility condition to stay in the relationship with the current partner, *r*_0__1_ = −0.604, *SE* = 0.193, *z* = −3.133, *p* < 0.001 (the estimated values of maintaining relationship after the partner’s do-nothing choice were 3.07 and 2.46 controlling for gender in the low and high mobility conditions, respectively). Although we do not have a good explanation of this unexpected effect of mobility, it might have been due to the familiarity effect, which is an experimental artifact. As a necessary consequence of the experimental manipulation, participants in the low mobility condition interacted with the same partner more than those in the high mobility condition. Therefore, participants in the low mobility condition tended to rate their preference for an already-familiar partner more highly. Because of this possibility, we refrain from further interpreting this unexpected finding. There were no gender differences, *r*_02_ = 0.469, *SE* = 0.394, *z* = 1.189, *p* = 0.234.

**FIGURE 3:**
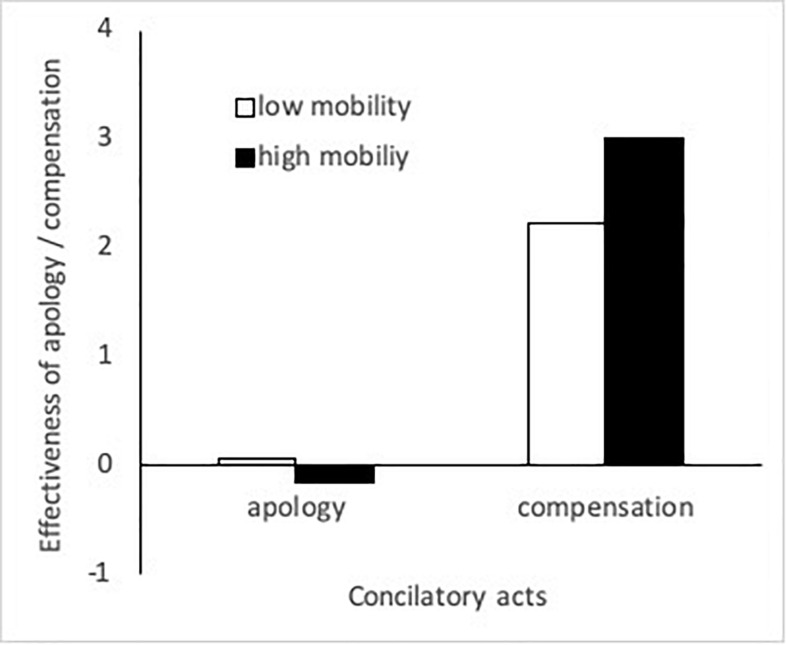
The effectiveness of apology and compensation on forgiveness in the high- and low-mobility conditions, after controlling for gender (Study 3). The scores were calculated by subtracting the estimated intercepts (i.e., did nothing) from the estimated values of forgiveness after each reconciliation behavior (i.e., apology or compensation). The estimated values were calculated by entering the average score (0.61) into each gender term.

Most central to our hypothesis, as predicted, participants in the high mobility condition were more willing than those in the low mobility condition to forgive the partner’s transgression, when the partner compensated relative to when the partner did not, *r*_11_ = 0.765, *SE* = 0.324, *z* = 2.363, *p* = 0.009 (see right-hand bars in Figure 3). There were no gender differences in the effect of compensation, *r*_12_ = −0.497, *SE* = 0.556, *z* = −0.894, *p* = 0.371.

Consistent with Studies 2a and 2b (although contrary to our original hypotheses), there were no differences between the high and low mobility conditions in the relative effect of apology over do nothing, *r*_21_ = −0.221, *SE* = 0.163, *z* = −1.355, *p* = 0.175 (though the result was in the expected direction with the low mobility participants showing a slight tendency to stay in the relationship with an apology: see the left-side bars in Figure 3). There were no gender differences, *r*_22_ = −0.539, *SE* = 0.351, *z* = −1.534, *p* = 0.125 (women showing a slight tendency to stay in the relationship with an apology).

In sum, the results generally supported our hypotheses. Participants in the high-mobility condition were more likely than those in the low-mobility condition to forgive the current partner’s transgression if compensation was given relative to non-compensation. Our findings suggest that the experimentally induced partner mobility shifted the preference for a reconciliatory behavior of an interaction partner. When people expect to switch partners often, they prefer a concrete compensation from the partner. When people expected to stay with the same partner, they showed a slight tendency toward preferring an apology. The non-significant effect of the low mobility on apology could partly be due to the contexts of the current experiment. In our experiment, low mobility participants did not expect to interact with the same partner for a long period of time—there still was a 20% chance of the partner changing in the next round. If the probability of the partner change had been much smaller and the length of interaction had been much longer (longer than the experimental session of one hour), we might have observed a stronger effect on apology. Related to this point, there are other limitations in the current study due to the experimental settings. For example, participants were not allowed to provide both verbal apology and compensation—an effective combination conciliatory gestures (Ohtsubo and Watanabe, 2009; Ohtsubo, 2020). This might have undermined the external validity. Moreover, since this experiment was conducted only in Japan, a different pattern might be shown in other cultural contexts such as the United States. It is important to examine these issues in the future.

## A *post hoc* Power Analysis

Before moving on to the general discussion, we note that we conducted a series of *post hoc* power analyses of Studies 1 and 2 (we did not conduct a comparable *post hoc* power analysis for Study 3 due to its complicated design; see also footnote 4 for a discussion of the stability of the findings). Our primary findings were that residential mobility boosts the effectiveness of compensation, whereas it impairs the effectiveness of verbal apology. As reported in each section, for compensation, the effect size ranged from small to medium (i.e., partial η^2^ = 0.03 [*f* = 0.18] in Study 1, partial *r*^2^ = 0.027 in Study 2a, and partial *r*^2^ = 0.027 in Study 2b [simple slope tests]). As for apology, the effect sizes of mobility fluctuated across studies (i.e., partial η^2^ = 0.13 [*f* = 0.39] in Study 1 vs. partial *r*^2^ = 0.001 and 0.002 in Studies 2a and 2b). We used G^∗^power 3.1 to obtain *post hoc* power on the basis of the observed effect sizes and sample sizes (Faul et al., 2009). The estimated powers were the following: 1.00 in Study 1, 0.61 in Study 2a, and 0.67 in Study 2b for the mobility-compensation effectiveness association; and 1.00 in Study 1, 0.07 in Study 2a, and 0.10 in Study 2b for the mobility-apology effectiveness association. These *post hoc* power analyses imply that we did not have sufficient sample sizes in each study. Future studies should address this issue.

## General Discussion

The purpose of the present study was to test the socio-ecological hypothesis of reconciliatory tactics: apology is more likely to lead to reconciliation in a residentially stable context, whereas compensation is more likely to lead to reconciliation in a residentially mobile context. Evolutionary psychologists have investigated the reconciliatory processes assuming that its function is to preserve valuable relationships (McCullough, 2008; McCullough et al., 2014; Ohtsubo and Yagi, 2015). From this perspective, the present study demonstrated how socio-ecological constraints could influence the value of a relationship and the effectiveness of reconciliatory behaviors. Specifically, we predicted that the effect of apology would be larger for people in residentially stable environments than for people in residentially mobile environments, while the effect of compensation would be larger for people in residentially mobile environments than for people in residentially stable environments. Four studies, each employed different conceptualization of mobility, partly confirmed the prediction. Study 1 (cross-national comparison) showed that apologies were received more favorably in Japan (i.e., a less mobile country) than in the United States (i.e., a highly mobile country), while compensation was received more favorably in the United States than in Japan. An offense was more likely to be forgiven in Japan than in the United States if the offender offered an apology, whereas an offense was more likely to be forgiven in the United States than in Japan if the offender offered a compensation. Studies 2a and 2b (within-country comparison) showed that compensation was more effective to appease those who had experienced more moves than those who had experienced less moves, though there was no significant effect of mobility on the effectiveness of apology. Experimentally manipulating mobility in the laboratory, Study 3 showed that mobility (i.e., frequent partner change) undermined the overall cooperativeness in transient “societies” and increased victims’ demands for compensation. Again, Study 3 failed to find the effect of residential mobility on apology.

### The Socio-Ecological Hypothesis of Reconciliation

Evolutionary psychologists have recently suggested that reconciliation occurs because breaking up valuable relationships invites more cost than preserving them (McCullough, 2008; Burnette et al., 2012; McCullough et al., 2014; Ohtsubo and Yagi, 2015; Smith et al., 2020). When the relationship is valuable and the exploitation risk is low, people engage in reconciliation for the benefit it affords them. From this perspective, previous studies have examined how each relationship value or individual act affects the occurrence of reconciliation, and successfully provided empirical evidence supporting their hypotheses.

In addition to each individual or relationship characteristic, the present study suggests that the reconciliation process is also constrained by the social ecology surrounding individuals. Specifically, in residentially stable environments, it is more adaptive for people to take a longer time-perspective and to let some mishaps go in order to preserve good relationships (Hruschka and Henrick, 2006). On the other hand, in mobile environments, the cost of quitting the current relationship is relatively low, and thus people may apply a more stringent strategy—to quit the current relationship unless the partner provides a costly, credible signal of reconciliation (i.e., compensation). Our findings provide empirical evidence supporting at least the latter prediction; residential mobility could influence the effectiveness of compensation.

This socio-ecological framework is important because it means that the societal differences in reconciliation styles can be unpacked by a more objective, measurable socio-ecological factor than subjective psychological values (Oishi and Graham, 2010; Oishi, 2014). In the present study, we found a key to explain why compensation is more prevalent in the United States, which is one of the most residentially mobile countries, compared to Japan, which is a residentially stable country (Study 1). The difference in the prevalence of compensation is not attributable to culture, because the individual differences in residential moves (Studies 2a and 2b) and experimental manipulation (Study 3) influenced the preference for compensation. These results contradict explanations appealing to culturally shared values/norms, but rather are consistent with the socio-ecological explanation that emphasizes more objective environmental parameters.

In addition, it is noteworthy that the socio-ecological perspective allows us to directly test the environment-individual interaction. In Study 3, we created high-mobility vs. low-mobility “societies” in a laboratory, and examined how individuals behave in each. In particular, we assessed not only participants’ reactions to apology and compensation but also their cooperativeness and reconciliatory tactics. Study 3 suggests that the environmental variable may affect each of these elements, which are also mutually interlocked in a given society. Such dynamics between individuals and environments could not be examined without the socio-ecological perspective. We note that the importance of ecological factors has not been neglected in evolutionary psychology.

In sum, a distinctive feature of this approach was a multi-level demonstration of the effect of mobility and the combination of correlational and experimental approaches to test it. As a result, the present research succeeded in demonstrating that a socio-ecological factor (i.e., mobility) could explain both cross-national differences and individual differences in reconciliatory tactics, confirming the external validity of the effect of mobility on reconciliatory tactics.

### Effectiveness of Compensation in High-Mobility Environments

The present research provided clear evidence supporting the relationship between residential mobility and the effectiveness of compensation. In Study 1 and Studies 2a and 2b, residentially mobile people (i.e., Americans and frequent movers) were more likely to be appeased by compensation than less mobile people (i.e., Japanese and less frequent movers). Study 3 also showed that the residentially mobile situations forced people to be more forgiving of the offenders who provided compensation than the residentially stable situations. Overall, all four investigations which examined societal, individual, and situational differences supported our hypothesis that compensation is a more effective way to promote reconciliation in high-mobility environments.

Although the four studies confirmed our hypothesis, the reason why compensation is effective is still unclear. Compensation means two things for victims in mobile environments. First, as we have already noted, as it is a costly conciliatory act, it communicates the perpetrator’s sincere intention to victims (Nakayachi and Watabe, 2005; Ohtsubo and Watanabe, 2009; Ohtsubo et al., 2018). In addition, as another possibility, compensation allows victims to immediately recoup their damage at least partially (Darley and Pittman, 2003; Desmet et al., 2011).

Both of the above two aspects of compensation can be important for victims in mobile environments. First, as we saw in Study 3, general cooperation rate tends to be low in mobile environments. Therefore, people in mobile environments must be cautious in assessing credibility of a transgressor’s reconciliatory signals. As a result, they may come to prefer more credible signals (e.g., compensation) than less credible signals (e.g., merely saying “I am sorry”). Consistent with this thesis, recent studies (Yamada et al., 2017; Komiya et al., 2019) have uncovered that even outside the context of reconciliation, people in mobile environments (e.g., United States) tend to send costly signals of commitment within intimate relationships (e.g., romantic gifts) than those is relatively stable environments (e.g., Japan). Second, in highly mobile societies, people may develop a less intimate form of relationship (i.e., exchange relation) with many short-term partners. In exchange relationships, people tend to use a shorter time perspective (Lydon et al., 1997). If the victim cannot expect a long-lasting relationship with his/her perpetrator, it is wise for him/her to ask the perpetrator to provide compensation as soon as possible.

Since this was the first study to examine the effect of mobility on effectiveness of compensation, it is still unclear which aspect of compensation (or joint presence of the two aspects) is more important to account for the observed effect. Although these two factors seem to be deeply intertwined, it may be possible to experimentally separate them. Future studies must disentangle these two accounts of the compensation effect.

### Effectiveness of Apology in Low-Mobility Environments

We predicted that people in stable environments would respond more favorably to a non-costly form of apology than those in less stable environments. In residentially stable environments, established relationships can be considered as valuable commodities because it is difficult and costly to cultivate new relationships. Therefore, victims are better off staying in the established relationships with a transgressor (and seeing what he/she will do next) than immediately withdrawing from the relationship. This prediction was partly supported. A cross-national comparison (Study 1) showed apologies were more effective in maintaining relationships in a residentially stable country (i.e., Japan) than in a less residentially stable country (i.e., United States). However, this pattern failed to reach the conventional level of statistical significance in an experimental study (Study 3). The comparable pattern was not found in a within-nation comparison (Studies 2a and 2b). The effectiveness of apology did not depend on the number of moves participants experienced.

The simplest explanation might be culture (here defined as “explicit and implicit patterns of historically derived and selected ideas and their embodiment in institutions, practices, and artifacts” Adams and Markus, 2004, p. 341). That is; relationship concern might be shared among members of a given culture to a similar extent, and might not vary across individuals or contexts with different rates of residential mobility.

Or, apology’s effectiveness might be based on social norms. We assumed that a verbal apology would be enough to signal sincerity and preserve relationships, because individuals in a stable community rarely betray each other and exploitation risk is estimated as being low. However, verbal apology might not unconditionally signal sincerity even in a stable community. In a stable community, if someone repeatedly exploits community members and offers perfunctory apologies, he/she will be sooner or later ostracized, whereas this is not the case in a mobile country due to the ease of finding new partners (Wang and Leung, 2010; Wang et al., 2011). It has been shown that such informal sanction against dishonesty serves to keep apparent cheap talk (e.g., merely saying “I am sorry”) credible (Ohtsubo et al., 2018). Without such a social norm to enhance credibility of apologies, even residentially stable individuals might not forgive apologizing transgressors. In other words, the informal sanction system might be critical for the effectiveness of verbal apology especially in a stable community, lowering the risk of being exploited. Regardless of which explanation is valid (or other explanation is required), further studies are needed to explain why verbal apologies tended to be more favorably perceived in Japan than in United States.

### Theoretical Implications

#### Culture and Reconciliatory Acts

Cross-national research has typically attributed the difference in the endorsement of reconciliatory acts to cultural differences. In East Asian countries such as Japan, reconciliatory acts were more highly favored than in Western countries such as the United States because they represent the concern for harmonious interpersonal relationships (Itoi et al., 1996; Hook et al., 2009; Ohbuchi et al., 2009). More directly, Fehr and Gelfand (2010) found that those who emphasized the independent self-construal were appeased more by compensation, while those who emphasized relational or collective self-construal were appeased more by other cost-free forms of apology. Their finding was consistent with the findings of Studies 1 and 2b in the present research. Especially, Study 2b showed that Caucasian Americans evaluated compensation more favorably than non-Caucasian Americans (mostly Asian Americans) irrespective of individual residential mobility, confirming that culture plays an important role on determining which type of conciliatory acts is effective in reconciliation.

In contrast, we found that at least a certain type of reconciliatory acts such as compensation could be better explained by a socio-ecological hypothesis rather than by a cultural explanation. This is because cultural, individual, and situational differences of residential mobility are consistently associated with the variation of favoring compensation. This fact suggests the importance of social ecology to explain reconciliatory acts as well as cultural contexts.

More generally, unlike cultural psychological approach, the socio-ecological approach is applicable to within-culture individual differences and situational influences. These features are shared by another growing discipline called human behavioral ecology, which emphasizes human phenotypic plasticity and encompasses between-populations, within-population, and within-individual variations (Nettle et al., 2013). Nonetheless, as human behavioral ecology was pioneered by anthropologists, it tends to employ correlational studies and to not cover social psychological topics, such as reconciliation. The present research is thus unique to emphasize phenotypic plasticity in social psychological phenomena and to employ the experimentation to test the causal effect of a socio-ecological factor.

#### Communal Cooperation

As a socio-ecological research, this is the first study to examine effects of a socio-ecological factor, residential mobility in particular, on reconciliatory tactics. Indeed, a large body of research on residential mobility-stability and cooperation has been accumulated since the 1970s. For example, early research has repeatedly shown that rural residents are more helpful than urban residents (Milgram, 1970; Korte and Kerr, 1975; Korte et al., 1975; Steblay, 1987 for review). More recently, Oishi et al. (2007b, 2009) found that residential stability promotes pro-community actions. Lun et al. (2012) further reported that non-movers prefer individuals who selectively cooperate with in-group members to unconditional cooperators, whereas this is not the case with frequent movers.

In a sense, the present research is positioned as a logical extension of these findings. The high level of cooperation in residentially stable societies fosters a social norm of unconditional forgiveness. On the other hand, the relatively low level of cooperation in residentially mobile societies requires a costly act of reconciliation. The present study demonstrated socio-ecological differences in reconciliation tactics as a consequence of communal cooperation and contributed to developing a more elaborated model of communal cooperation.

### Limitations and Future Directions

Although the present study is the first study to examine the socio-ecological hypothesis of reconciliatory tactics, there are several limitations. First, the present study fully supported the hypothesis about compensation, but failed to support the hypothesis about apology. As we have already discussed, the lack of social norms which support informal sanction system might attenuate the influence of residential mobility on the effectiveness of apology. Future studies can confirm this prediction by manipulating the chance to develop social norms, such as enabling people to observe other people’s behaviors. This examination would clear whether the cultural-value explanation (collectivism) or the socio-ecological explanation (informal sanction system) or both is important to explain the difference of reconciliation at the societal level.

Second, though it is not our main concern, we found unexpected patterns for the baseline. Specifically, Americans (i.e., people living in a high-mobility environment) were more likely than Japanese (i.e., people living in a low-mobility environment) to maintain relationships with offenders who provided no conciliatory acts in Study 1, whereas participants under the low mobility condition reported greater willingness to maintain relationships with the same do-nothing partner than participants under the high mobility condition (Study 3). Although possible reasons why we obtained these patterns were discussed in each section, they remain speculation. It would be promising to investigate which factors determine the level of forgiveness in each socio-ecological environment.

Third, we spotted another seemingly contradictory pattern. Irrespective of the level of mobility, offenders in Study 3 showed no clear preferences for any particular reconciliatory tactics. Combined with the attenuated effect of mobility on victims’ preferences for apology, this null effect suggests that the victims’ preferences for compensation under highly mobile environments might have the rein on the extant cultural differences. Regardless of the mobility of the environments, perpetrators might first attempt to see their victims’ responses by offering non-costly apologies. In a mobile environment, as the victims are highly demanding, the perpetrators may eventually learn that only compensation would work. On the other hand, in a stable environment, the perpetrator might learn that a non-costly apology would work as well as compensation (i.e., a more costly form). If this is the case, the only required psychological mechanism is the victim’s preference for compensation in mobile environments. Although the scope of the present study did not encompass perpetrators’ behavioral tendencies or relevant social norms, in future studies, it will be worthwhile to investigate these aspects by modifying the present study.

Finally, there are some measurement issues. First, the present study used a single-item or two-items to measure the reconciliatory tendency, which might lower the validity and the reliability. Thus, it would be desirable to replicate the present findings using a multiple-item measure of reconciliation. In addition, we admit that willingness to maintain the relationship is not the same as forgiveness, which is the theme of this special issue. Although, as we argued in the introduction section (especially section “Evolutionary Approach to Reconciliation and Forgiveness”), we believe that willingness to maintain the relationship is closely connected with forgiveness, future research needs to include forgiveness measures to directly examine the conceptual relationship between forgiveness and willingness to maintain the relationship.

## Conclusion

The present research shows that reconciliatory tactics, apology and compensation, can be constrained by socio-ecological factors such as residential mobility. Evolutionary psychology has accumulated a massive body of work on various social behaviors (e.g., emotion recognition, mate selection), including reconciliatory tactics. At this point, however, the findings from evolutionary psychology have not investigated the ecological influence on reconciliation. Through investigation of societal, individual, and situational differences, the current research provides empirical evidence supporting that residential mobility fosters the preference for compensation. Although the present findings showed that residential mobility at an individual level did not influence the effectiveness of apology, its asymmetry suggests a new possibility that the effectiveness of apology could be based on social or cultural norms. More generally, the current research demonstrates that the socio-ecological approach presents a promising empirical and theoretical framework through which psychological scientists can integrate divergent findings from evolutionary psychology, cultural psychology, and beyond.

## Data Availability Statement

The datasets for this study can be found in the OSF: https://osf.io/g8cux/.

## Author Contributions

AK, HO, and MW contributed conception and design of the study. AK, HO, MW (Study 1 and Study 3), and YO (Study 2a) were involved in data collection in Japan. AK, YM (Study 1), and SO (Study 2b) collected data in the United States. AK and SO performed the statistical analysis. AK wrote the first draft of the manuscript. YO and SO wrote the sections of the manuscript. All authors contributed to manuscript revision and read and approved the submitted version.

## Conflict of Interest

The authors declare that the research was conducted in the absence of any commercial or financial relationships that could be construed as a potential conflict of interest.

## Footnotes

1 For the additional analysis to test the fitness of the overall model, the contrast analysis (Rosenthal et al., 2000) see Supplementary Material.

2 Because this study was re-analysis using unpublished data which was conducted for another purpose (Komiya et al., 2018), there was another variation for each scenario (i.e., two versions of each scenario; see Supplementary Material). Since the variations did not significantly influence the effect of moving experiences on apology/compensation, we did not include this variation as an independent variable in the following analyses. This variation was not included in Study 2b.

3 We excluded the intentional offenses from this analysis for two reasons. First, the options of “not to share” and “compensation” (namely, choosing compensation after intentional offenses) was completely irrational in this experiment, as the player would be better off choosing “share” initially (60 JPY), instead of making this decision (30 JPY). Accordingly, there were few such cases (only one in 229 intentional offenses). Second, choosing “apology” after “not to share” might have been a dishonest signal, as anyone was able to send this signal without incurring any cost. If these were deceptive signals, the apology-after-intentional-offense should have been more frequently used in the less cooperative condition (i.e., the high mobility condition) than in the more cooperative condition (i.e., the low mobility condition). Consistent with this prediction, the apology-after-intentional-offense rate was slightly higher in the high mobility condition (0.80, *SD* = 0.30) than in the low mobility condition (0.63, *SD* = 0.39), *t*(55.09) = 1.94, *p* = 0.057, *d* = 0.48. As we are interested in the more or less honest conciliatory acts, we excluded the cases of intentional offense.

4 Some participants (*n* = 13) never entered the apology stage because they never chose “trust” or their partners always chose “share” and were not affected by the “noise.” Therefore, those participants’ data were excluded from the multilevel-model analysis. According to Maas and Hox (2005) paper, Level 2 sample size of 100 produced unbiased estimates. Thus, our sample size (Level 2 = 85) was less likely to be sufficient to produce unbiased estimates. This issue should be addressed in the future.
