# Supplementary material for: Socio-Ecological Hypothesis of Reconciliation: Cultural, Individual, and Situational Variations in Willingness to Accept Apology or Compensation
Source: Front Psychol. 2020 Jul 23;11:1761. doi: 10.3389/fpsyg.2020.01761 (PMC7390922; doi:10.3389/fpsyg.2020.01761)
Supplement: Supplementary file 1 [file Data_Sheet_1.docx]

Supplementary Material

# Scenarios used in Study 1

*The order of the perpetrator’s behavior was fixed (a within-participant factor).

Musical Scenario

Kelly and Jessica decided to go to a musical. They have been very much looking forward to seeing it since the show is very popular and it is hard to get the tickets. On the day of the musical, Kelly did not show up however long Jessica waited and Jessica could not contact her. Since Kelly had the tickets for both of them, Jessica could not enter the theater. Finally Kelly showed up 1 hour after the musical has started and they missed the first part of the musical.

**a) AC condition (Apology + Compensation):**

Kelly said, “I am really sorry [*gomen nasai* in the Japanese version] for being late.” and paid Jessica for her ticket.

**b) NC condition (No Apology + Compensation):**

Kelly did not apologize for being late at all and paid Jessica for her ticket.

**c) AN condition (Apology + No Compensation):**

Kelly said, “I am really sorry for being late.” but she did not pay Jessica for her ticket.

**d) NN condition (No Apology + No Compensation):**

Kelly did not apologize for being late at all and did not pay Jessica for her ticket.

Travel Scenario

Erica and Sarah decided to go to a short trip by bus with a couple of other friends. They originally planned to meet at the university and go together. A day before their departure, they changed their plans and decided to meet at the bus terminal since one of their friends who lives near the bus terminal wanted to join them. However, on the day of their departure, Sarah didn’t show up, even past the time they were supposed to meet. Erica then realized that she forgot to tell Sarah that they changed their plan for where they would meet.

**a) AC condition (Apology + Compensation):**

Erica made a phone call to Sarah and said, “I am sorry I did not tell you about the change of our plan. We decided to meet at the bus terminal instead of the university. So please come to the bus terminal as soon as possible. I will pay you for the taxi.”

**b) NC condition (No Apology + Compensation):**

Erica made a phone call to Sarah and said, “We decided to meet at the bus terminal instead of the university. So please come to the bus terminal as soon as possible. I will pay you for the taxi.” And Erica did not apologize at all.

**c) AN condition (Apology + No Compensation):**

Erica made a phone call to Sarah and said, “I am sorry for not telling you that we have changed our plan. We changed our plan and decided to meet at the bus terminal, not at the university. So please come to the bus terminal as soon as possible.”

**d) NN condition (No Apology + No Compensation):**

Erica made a phone call to Sarah and said, “We changed our plan and decided to meet at the bus terminal, not at the university. So please come to the bus terminal as soon as possible.” Erica didn’t apologize at all.

Book Scenario

Craig borrowed Nick’s valued book from him. While reading the book, Craig tripped and spilled his coffee. Although he wiped it immediately, the book was clearly stained with coffee.

**a) AC condition (Apology + Compensation):**

Craig said, “I’m sorry for staining the book.” and bought a new book and returned it to Nick.

**b) NC condition (No Apology + Compensation):**

Craig did not apologize for staining the book at all but bought a new book and returned it to Nick.

**c) AN condition (Apology + No Compensation):**

Craig said, “I’m sorry for staining the book.” and returned the stained book.

**d) NN condition (No Apology + No Compensation):**

Craig did not apologize for staining the book at all and returned the book with the stain.

# Contrast Analysis (Study 1)

Rosenthal et al. (2000) recommend the contrast analysis for the research with focused hypotheses by stating that “Contrasts are statistical procedures for asking focused questions of data... Compared to diffuse or omnibus questions, focused questions are characterized by greater conceptual clarity, and the statistical procedure by greater statistical power...Contrast analyses yield both estimates of the magnitude of the effects investigated and the associated significance levels” (p. 1). Since we have a very focused prediction, we conducted the contrast analysis, as advocated by Rosenthal et al. (2000).

As stated in the main manuscript, our central prediction was that apology would be more effective than compensation in Japan, whereas compensation would be more effective than apology in the U.S. In contrast, we predicted no cultural differences in the neither apology or compensation condition and the apology and compensation condition. Therefore, our hypotheses were (i) both Japanese and Americans would be more likely to forgive the offender in the AN condition than in the NN condition (thus, -2 for the NN among Japanese and Americans), but the apology effect would be larger for Japanese than Americans (thus, +1 for the AN among Japanese, -1 among Americans), (ii) both Japanese and Americans would be more likely to forgive the offender in the NC condition than in the NN condition, but the compensation effect would be larger for Americans than Japanese (thus -1 for the NC condition among Japanese, +1 among Americans), and (iii) both Americans and Japanese would be more willing to forgive the offender in the AC condition than in the NN condition, and the size of the effect would not differ between U.S. and Japan (thus +2 for the AC condition both among Japanese and Americans). In sum, we used the following coding: For Japanese, the contrast was -2, 1, -1, and 2 for the NN, AN, NC, and AC conditions, respectively. For Americans, the contrast was -2, -1, 1, and 2 for the NN, AN, NC, and AC conditions, respectively. We conducted a simple regression, predicting the forgiveness score from the contrast code above. This analysis showed that the contrast fit the data very well, explaining 45.5% of the variance, *R*^2^ = .46, *b* = 0.76, *SE* = 0.04, β = .67, *t*(402) = 18.30, *p* < .001, 95% CI [.68, .84], suggesting that the obtained data fit our hypotheses well.

# Scenarios used in Study 2

*Because Study 2a was re-analysis using unpublished data which was conducted for another purpose (Komiya et al., 2018), there was another variation for each scenario (i.e., two versions of each scenario). Since the variations did not significantly influence the effect of moving experiences on apology/compensation, we did not include this variation as an independent variable in the analyses reported in the manuscript.

*Variations are in square brackets. This variation was not included in Study 2b.

*Unlike Study 1, conciliatory acts is a between-participant factor.

Book Scenario

One of your friends borrowed your prized book. The book of yours, which was written by a well-known novelist, was*in a like-new condition.* [*a signed first edition of the book; you will never get the same one again.*] However, while reading the book, your friend accidentally tripped and spilled his coffee. Although he wiped it immediately, the book is now clearly stained with coffee. After reading the book, your friend told you that your book was stained with coffee.

**a) Apology:** Your friend said, “I’m sorry for staining the book.” and returned the stained book.

**b) Compensation:** Your friend did not apologize for staining the book at all but bought a new book and returned it to you.

Travel Scenario

You and one of your friends heard there was a baseball game in the local stadium, and bought a pair of tickets, which was a good bargain. Both of you looked forward to watching the game because that was *the first of three consecutive games of your favorite team in the stadium.*[*the last game of your favorite team in the stadium*.] On the day of the game, you waited and waited, but the friend did not show up. Also, you could not contact him/her. Because your friend had both your and his/her tickets, you could not enter the stadium. Your friend ended up arriving two hours late, so you and your friend missed the baseball game.

**a) Apology:** Your friend said, “I am really sorry for being late.”

**b) Compensation:** Your friend did not apologize for being late at all, but bought a new ticket for the next game (*where your favorite team would appear* [*where your favorite team would not appear*]) for you in the stadium.

# The procedure of the noisy trust game (in Study 3)

First, both the investor and the responder received an initial endowment of 30 JPY (approximately 0.3 USD). The investor first decided whether to entrust her/his endowment (30 JPY) to the responder. If the investor chose “not to trust,” both players would finish with 30 JPY on the round. If the investor chose “to trust,” the endowment was tripled and transferred to the responder. The responder then decided whether to equally share the increased resource of 120 JPY (= the responder’s initial endowment of 30 + transferred 90) with the investor. If the responder chose “to share,” both players would have a chance to receive 60 JPY. If the responder did not share the resource, the responder would finish with 120 JPY and the investor would receive nothing (i.e., the responder betrayed the investor’s trust).

If and only if the responder decided to share, he/she played a card game for a “noise.” In the card game, the responder chose one card from three cards (one of the three cards was a “failure” card; others were “success” cards) shown on the computer display. If the responder chose the “failure” card, he/she failed to choosing “to share” (i.e., the responder took all 120 JPY leaving nothing for the investor). If the responder chose a “success” card, both players received 60 JPY.
